# Supplementary material for: Transposable element RNA dysregulation in mutant KRAS(G12C) 3D lung cancer spheroids
Source: bioRxiv. 2023 Feb 28:2023.02.27.530369. Preprint. [Version 1] doi: 10.1101/2023.02.27.530369 (PMC10002638; doi:10.1101/2023.02.27.530369)
Supplement: supplement 1 [file NIHPP2023.02.27.530369v1-supplement-1.pdf]

bioRxiv preprint doi: https://doi.org/10.1101/2023.02.27.530369; this version posted February 28, 2023. The copyright holder for this preprint (which was not certified by peer review) is the author/funder, who has granted bioRxiv a license to display the preprint in perpetuity. It is made available under aCC-BY 4.0 International license.

SUPPLEMENTARY FIGURE

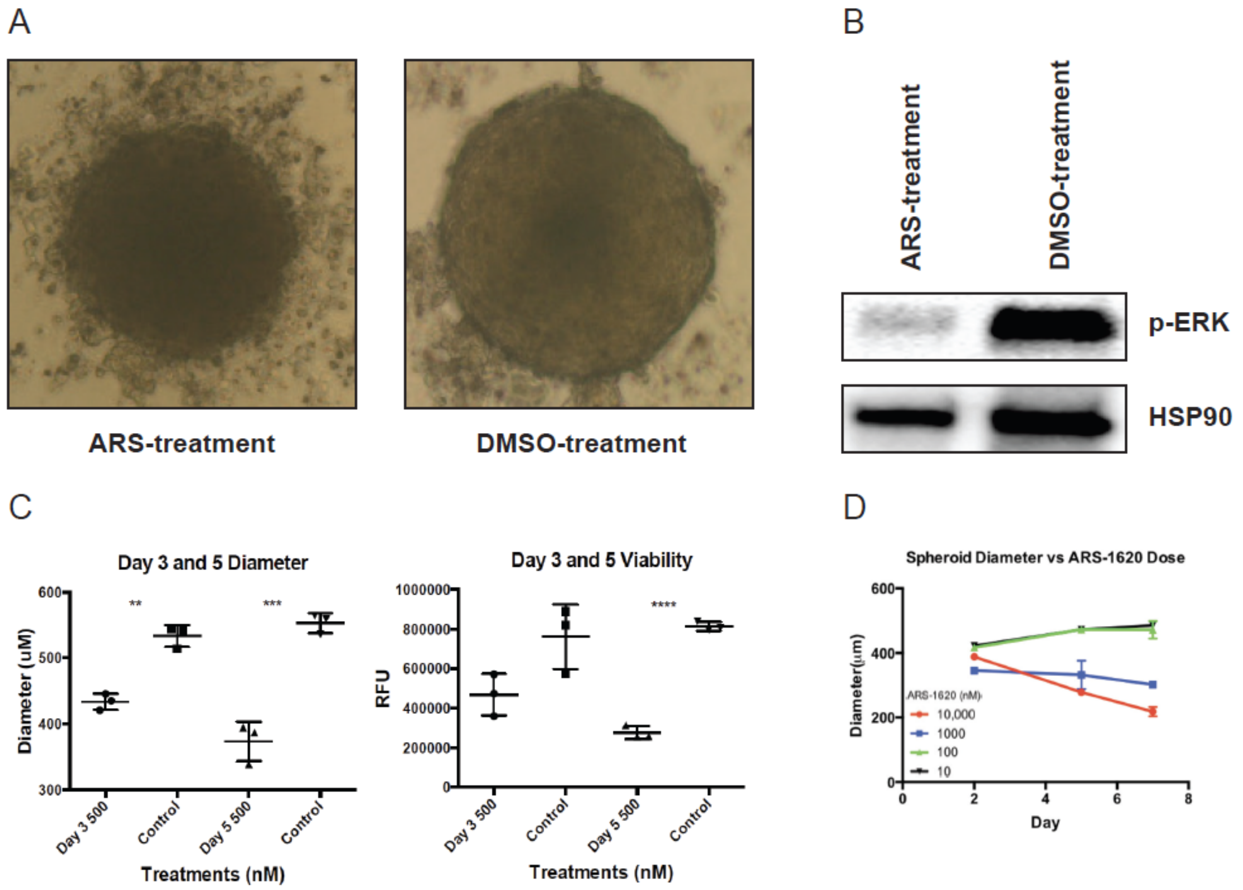

Figure S1.

A. H358 3D lung cancer spheroids treated with ARS or DMSO. **B**. Western blot for p-ERK and HSP90 using H358 3D lung cancer spheroids treated with ARS or DMSO. **C.** Diameter measurements (in micrometers) (left plot) and cell viability (Cell Titer-Glo® luminescent cell viability in relative fluorescence units) (right plot) of H358 3D lung cancer spheroids treated with ARS or DMSO after 3 or 5 days of treatment (500 nM ARS-1620 or DMSO). **D.** Diameter measurements (in micrometers) of H358 3D lung cancer spheroids treated with different concentrations of ARS-1620 (nM) for 7 days.
